# Rectal Arteriovenous Malformation Mimicking a Submucosal Tumor: Successful Long‐Term Outcome After Transcatheter Arterial Embolization With N‐Butyl‐2‐Cyanoacrylate‐Lipiodol

**DOI:** 10.1002/deo2.70303

**Published:** 2026-02-21

**Authors:** Hiroshi Baba, Masakazu Hirakawa, Takeshi Oda, Hiromu Hidaka, Masayuki Hirata, Sho Kakinouchi, Yasuko Tamaru, Yuji Abe, Yoshiki Asayama

**Affiliations:** ^1^ Department of Radiology Nakatsu Municipal Hospital Oita Japan; ^2^ Department of Radiology Kyushu University Beppu Hospital Oita Japan; ^3^ Department of Radiology National Hospital Organization Beppu Medical Center Oita Japan; ^4^ Department of Gastroenterology Nakatsu Municipal Hospital Oita Japan; ^5^ Department of Radiology Oita University Faculty of Medicine Oita Japan

**Keywords:** gastrointestinal arteriovenous malformations, N‐butyl‐2‐cyanoacrylate, rectal arteriovenous malformations, submucosal tumors, transcatheter arterial embolization

## Abstract

Rectal arteriovenous malformations (AVMs) are rare vascular anomalies that pose diagnostic challenges because they may resemble submucosal tumor–like lesions. A 54‐year‐old man presented with recurrent hematochezia, and a colonoscopy revealed a protruding lesion near the dentate line. Contrast‐enhanced computed tomography and angiography confirmed a rectal AVM classified as Cho type IIIb/Yakes type II. Transcatheter arterial embolization with N‐butyl‐2‐cyanoacrylate–Lipiodol (NL) was performed. A rectal ulcer that developed 2 weeks after treatment was successfully managed with topical hydrocortisone. Follow‐up colonoscopy demonstrated progressive lesion regression without recurrence at 3 years. This case underscores that rectal AVMs may mimic submucosal tumors and require a multimodal diagnostic approach. Transcatheter arterial embolization with NL represents an effective therapeutic option.

## 1 Introduction

Gastrointestinal arteriovenous malformations (AVMs) are a rare cause of intestinal hemorrhage [1], most frequently occurring in the right colon (37%) and small intestine (43%) while being uncommon in the rectum (8%) [1]. Rectal AVMs may resemble ulcerative colitis–like conditions, such as mucosal congestion, edema, and erosion, or present as protruding lesions mimicking polyps or submucosal tumors [2]. The characteristic clinical manifestation is painless, recurrent bleeding, although some cases may progress to massive hemorrhage requiring urgent intervention [3]. Owing to the rarity of rectal AVMs, standardized treatment protocols remain undefined. This report presents a case of a rectal AVM resembling a submucosal tumor, incidentally identified during colonoscopy and successfully managed by transcatheter arterial embolization (TAE) with N‐butyl‐2‐cyanoacrylate–Lipiodol (NL), with favorable long‐term outcomes.

## 2 Case Report

**FIGURE 1:**
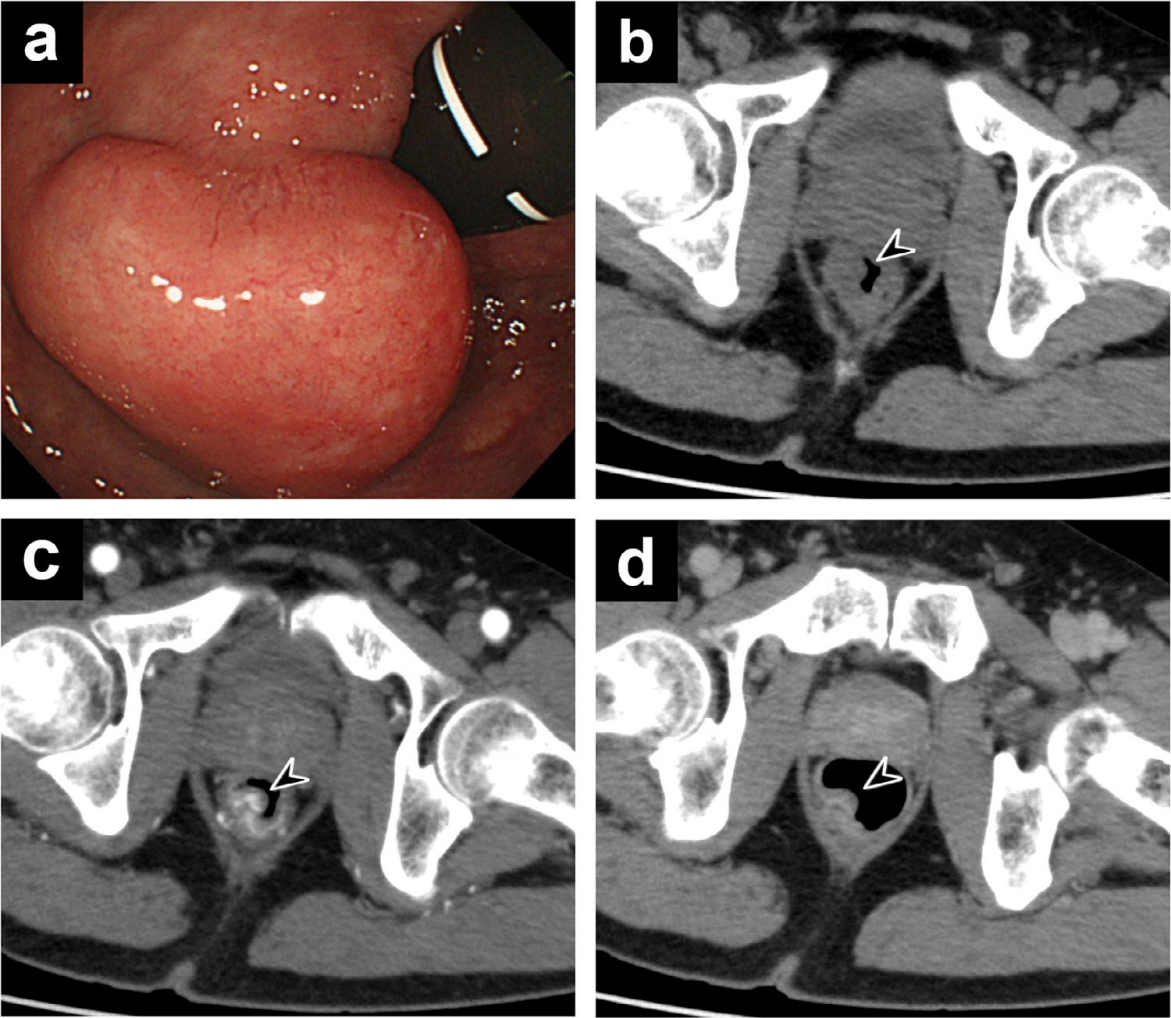
(a) Colonoscopy showing a submucosal tumor‐like protruding lesion in the lower rectum near the dentate line, with normal overlying mucosa. (b) Non‐contrast computed tomography showing a mass protruding into the lumen on the right side of the lower rectum (arrowhead). (c) Early‐phase contrast‐enhanced computed tomography demonstrating a hypervascular lesion with a cluster‐like vascular pattern (arrowhead). (d) Late‐phase image showing persistent contrast enhancement of the lesion (arrowhead).

A 54‐year‐old man with recurrent episodes of hematochezia presented for the first time after a positive fecal occult blood test result during a routine health check‐up. He had no history of surgery, comorbidities, or relevant family history. Blood tests showed mild anemia (hemoglobin: 11.8 g/dL; red blood cells: 4.91 × 10^6^/µL; mean corpuscular volume: 76.6 fL). Colonoscopy revealed a protruding lesion with a submucosal tumor‐like appearance near the dentate line in the lower rectum (Figure 1a). The mass had a diameter of 3 cm and smooth margins. The mucosal surface showed slight capillary dilatation without ulceration, and no pulsation was observed. A comprehensive assessment of the mass was conducted using computed tomography (CT) lesion characteristics, with Doppler endoscopy and evaluation with biopsy forceps scheduled after CT. Therefore, no assessment of lesion firmness or vascularity was performed at this time. On further evaluation, non‐contrast CT revealed a 3 cm intraluminal mass on the right side of the lower rectum (Figure 1b). Contrast‐enhanced CT suggested a rectal AVM with either the middle or inferior rectal artery as the feeding vessel and the inferior mesenteric vein (IMV) as the draining vessel (Figure 1c,d). Due to the size of the lesion, surgical resection was considered; however, as preservation of anal function was prioritized, TAE was planned following diagnostic angiography.

**FIGURE 2:**
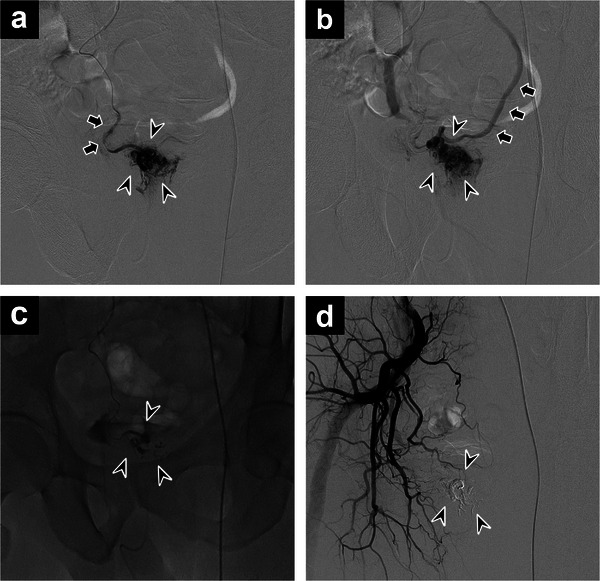
(a) Selective angiography of the right middle rectal artery (arrows) showing a nidus (arrowheads) supplied by multiple small feeding arteries. (b) The nidus (arrowheads) exhibiting early venous drainage into the inferior mesenteric vein (arrows). (c) Injection of a mixture of N‐butyl‐2‐cyanoacrylate and lipiodol, resulting in complete nidus filling (arrowheads). (d) Post‐embolization angiography of the right internal iliac artery confirming complete obliteration of the arteriovenous malformation, with stagnation of the embolic mixture within the nidus (arrowheads).

A 5‐Fr long sheath (Supersheath; Medikit, Tokyo, Japan) was inserted into the left femoral artery using the Seldinger technique. Diagnostic angiography was performed via the abdominal aorta, inferior mesenteric artery, bilateral common iliac arteries, and bilateral internal iliac arteries using a 4‐Fr pigtail catheter (JET BALANCE; Terumo, Tokyo, Japan) and a 4‐Fr shepherd's hook catheter (SHA; Medikit, Tokyo, Japan). Right internal iliac artery angiography revealed findings consistent with AVM in the lower rectum. Involvement of the middle or inferior rectal arteries was strongly suspected, as previously indicated by contrast‐enhanced CT. A 5‐Fr balloon catheter (Selecon MP Catheter II; Terumo, Tokyo, Japan) was positioned in the right internal iliac artery to allow flow control during embolization. A 2.0/2.8‐Fr microcatheter (BOBSLED ALLROUNDER; PIOLAX, Kanagawa, Japan) was then advanced, and selective contrast was administered to the right middle and lower rectal arteries. No involvement of the right inferior rectal artery was observed. Selective angiography of the right middle rectal artery revealed a fine nidus predominantly on the right side of the lower rectum with early venous drainage into the IMV, consistent with a diagnosis of rectal AVM (Cho type IIIb/Yakes type II; Figure 2a,b).

Following balloon dilation of the right internal iliac artery, contrast injected into the right middle rectal artery showed backflow into the right lower rectal artery before the nidus and subsequent drainage into the IMV. Because there was a high possibility of proximal embolization, embolization therapy without balloon occlusion was selected. The microcatheter was advanced close to the nidus, and embolization was performed using a 1:7 mixture of N‐butyl‐2‐cyanoacrylate and lipiodol without balloon occlusion. Injection was halted upon the appearance of backflow, and the microcatheter was withdrawn (injection volume: 0.8 mL; Figure 2c). Final angiography of the right internal iliac artery confirmed lipiodol stagnation at the nidus, complete obliteration of the AVM, and no new arterial flow to the lesion (Figure 2d).

**FIGURE 3:**
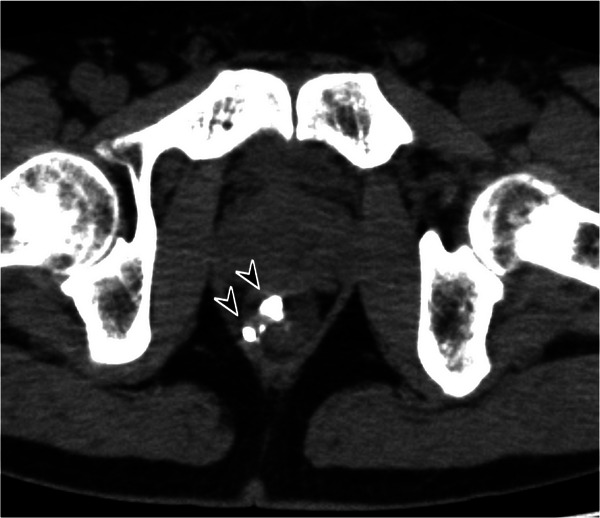
Non‐contrast computed tomography performed 10 days after transcatheter arterial embolization, showing lipiodol stagnation within the rectal arteriovenous malformation (arrowheads).

**FIGURE 4:**
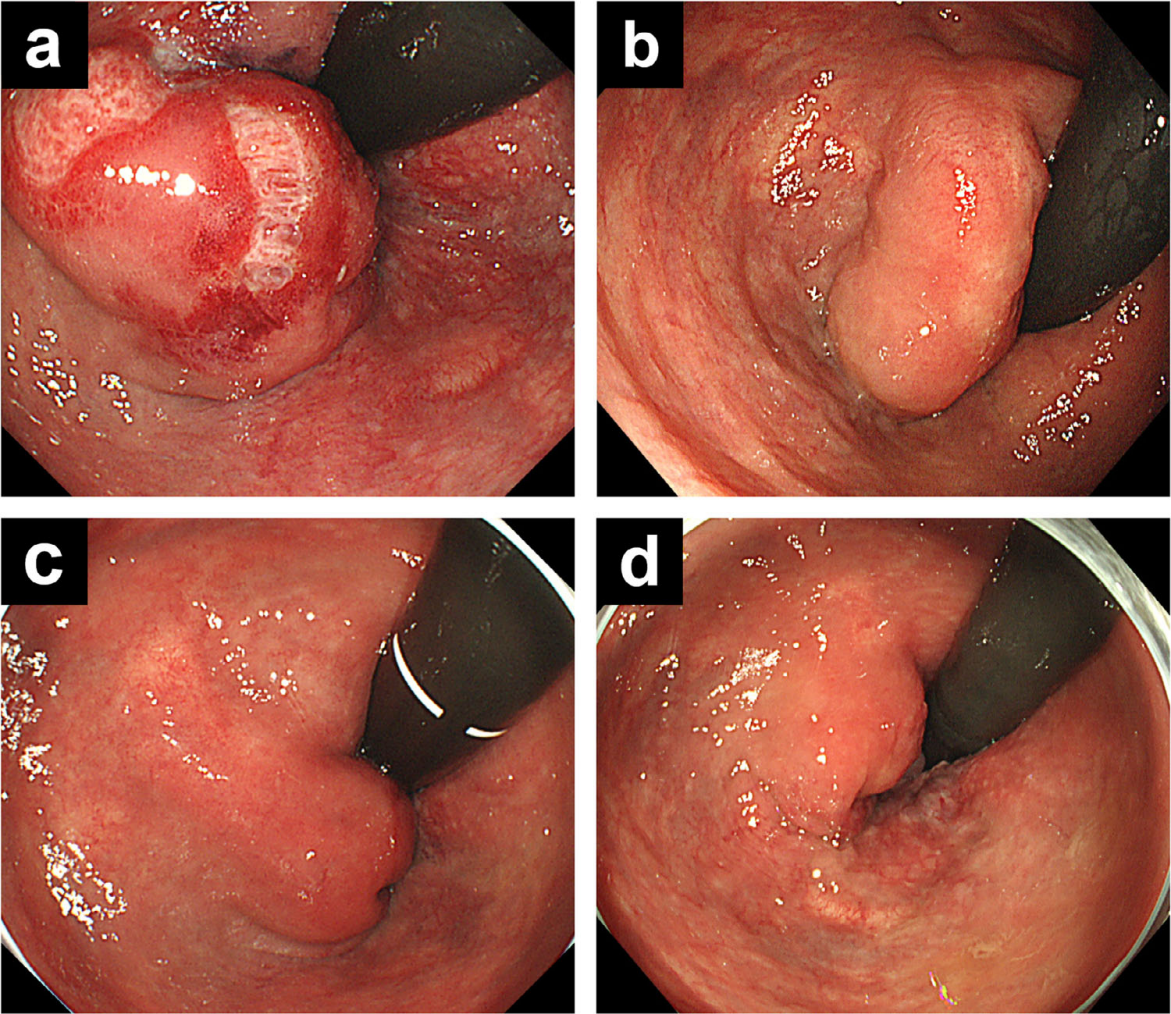
(a) Colonoscopy 14 days after embolization showing an ulcer on the mucosal surface of the lesion. (b) At 3 months, the ulcer has healed, and the lesion has decreased in size. (c) At 10 months, further reduction in lesion size is observed. (d) At 3 years, colonoscopy shows near‐complete resolution of the lesion.

CT performed 10 days post‐procedure showed lipiodol stagnation within the rectal AVM (Figure 3). No thrombi or embolic material were detected in the IMV or portal vein. Colonoscopy performed 2 weeks later showed ulcer formation, suggesting mucosal injury associated with TAE (Figure 4a). Because the lesion was located near the anus, topical hydrocortisone ointment was applied, and the bleeding was promptly controlled. Follow‐up colonoscopy was performed regularly. At 3 months, the ulcer had healed, and the lesion had decreased in size (Figure 4b). Follow‐up colonoscopy at 10 months and 3 years showed continued lesion regression, and the patient has remained in good physical condition after the treatment (Figure 4c,d).

## 3 Discussion

Rectal AVMs may present with clinical features resembling ulcerative colitis, such as mucosal congestion, edema, and erosion; alternatively, they may appear as protruding lesions mimicking polyps or submucosal tumors [2]. When the lesion presents as a submucosal tumor, as in this case, the overlying mucosa typically appears normal, making diagnosis by colonoscopy or barium enema difficult [4]. Even when mucosal abnormalities are present, biopsy findings often indicate chronic inflammation similar to enteritis [2]. Consequently, the diagnostic accuracy of colonoscopy for colorectal AVMs is limited, and although angiography is highly accurate at diagnosing this condition, its invasiveness facilitates a multimodal diagnostic approach [5]. In such cases, contrast‐enhanced CT may aid detection, while MRI can demonstrate the nidus as a flow void [2], and color Doppler endoscopic ultrasonography has also been reported to be useful [6]. In patients with recurrent painless lower gastrointestinal bleeding, gastrointestinal AVMs should be considered, and evaluation should extend beyond routine endoscopy.

Due to the rarity of the condition, there are no established size‐based criteria for selecting surgery in the context of treating rectal AVMs. Although surgical resection was historically the primary treatment for rectal AVMs, recent reports have shown that endovascular embolization is an effective alternative [2, 4, 6]. In addition, for lesions in the lower rectum, their surgical removal is accompanied by an increased risk of impairing the function of the anal sphincter and thus culminating in a reduction in postoperative quality of life. Endovascular embolization was selected in the present case, with favorable clinical outcomes. However, incomplete embolization carries a potential risk of recurrence.

There is a wide range of embolic agents, including metallic coils, gelatin particles, microspheres, polyvinyl alcohol, Onyx, and NL [4]. NL was selected because it enables rapid, permanent occlusion with deep nidus penetration, which is particularly advantageous in high‐flow arteriovenous shunts. Moreover, compared with non‐adhesive liquid embolic agents such as Onyx, NL allows shorter injection and fluoroscopy times, although meticulous technique is required to avoid non‐target embolization [7].

The Cho and Yakes classifications are angiographic systems that categorize AVMs based on the morphology of the arteriovenous shunt and the venous outflow pattern, both of which are relevant to treatment strategy [8, 9]. In the present case, angiography demonstrated multiple minute shunts between small arteries and small veins, forming a complex vascular network with venous drainage. Based on these findings, the lesion was classified as a Cho type IIIb and Yakes type II AVM. In this type, embolization of the feeding arteries alone may promote collateral vessel development or result in recanalization. Therefore, adequate embolization of the nidus itself is essential. Accordingly, we used NL to achieve complete nidus packing. Although balloon‐assisted flow control was initially attempted to prevent venous migration, retrograde flow was observed, raising concern for proximal embolization; therefore, balloon occlusion was not employed. Careful control of embolic distribution is critical to avoid both venous migration and proximal arterial obstruction.

Complications of TAE for rectal AVMs, including ulceration and stenosis, have been reported [5]. A rectal ulcer occurred 2 weeks after treatment in the present case, and given an overall complication rate of approximately 10% [10], careful follow‐up is required.

In conclusion, rectal AVMs may present as submucosal tumor–like lesions, limiting the diagnostic accuracy of endoscopy alone and carrying a risk of massive hemorrhage after biopsy. In patients with recurrent hematochezia or unexplained anemia, a vascular etiology should be suspected, and thus a biopsy should be avoided in favor of a multimodal diagnostic approach, including contrast‐enhanced CT and Doppler endoscopic ultrasonography when available. In this case, TAE with NL achieved complete nidus obliteration while preserving anorectal function.

## Author Contributions

All authors contributed equally to manuscript preparation and review.

## Funding

The authors received no specific funding for this work.

## Consent

Written informed consent was obtained from the patient for publication of this case report and accompanying images.

## Conflicts of Interest

The authors declare no conflicts of interest.
